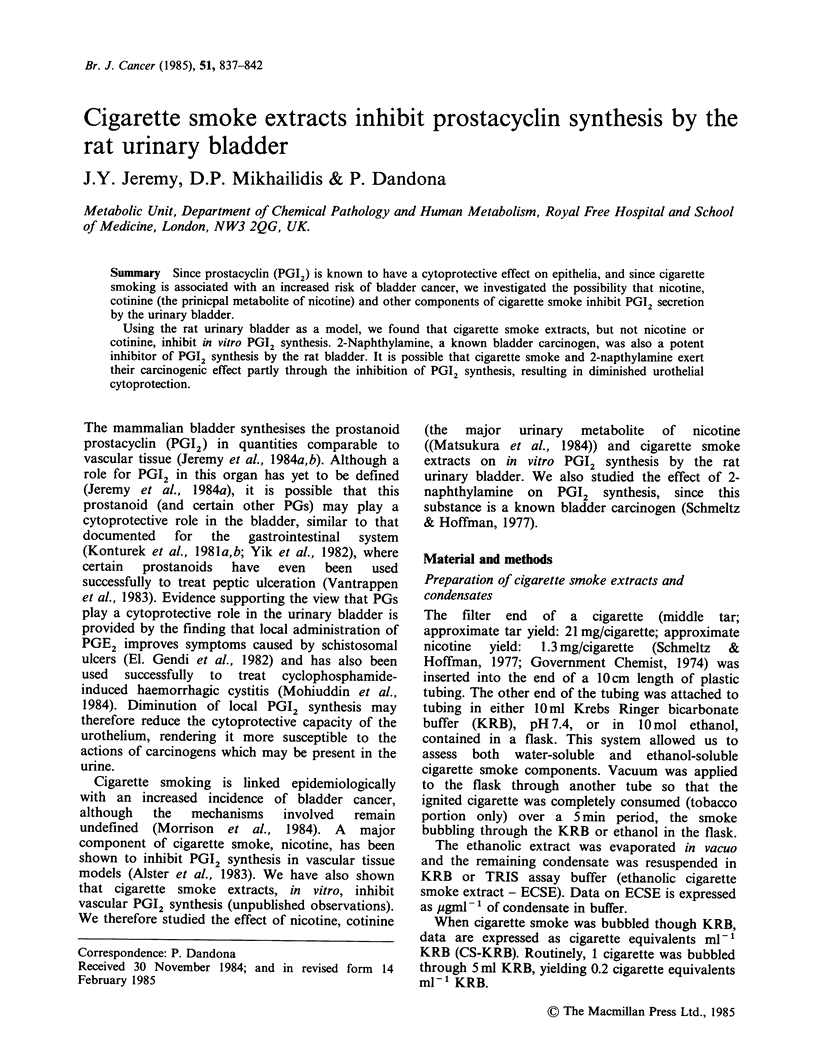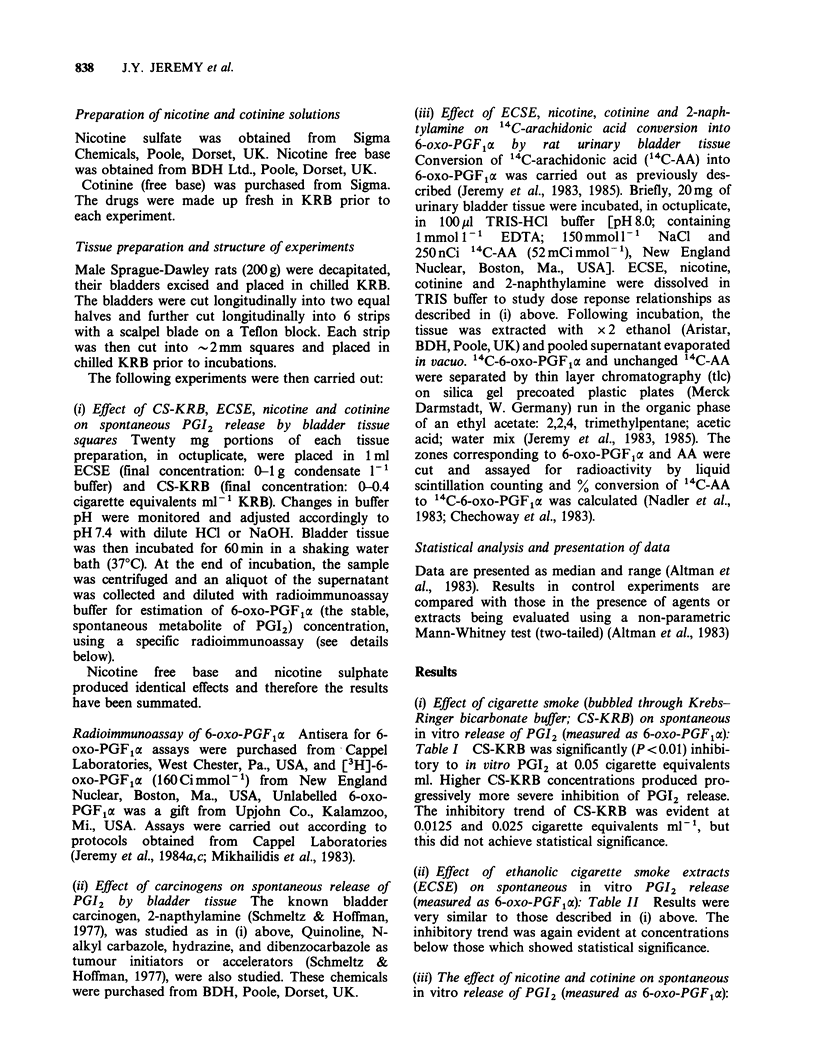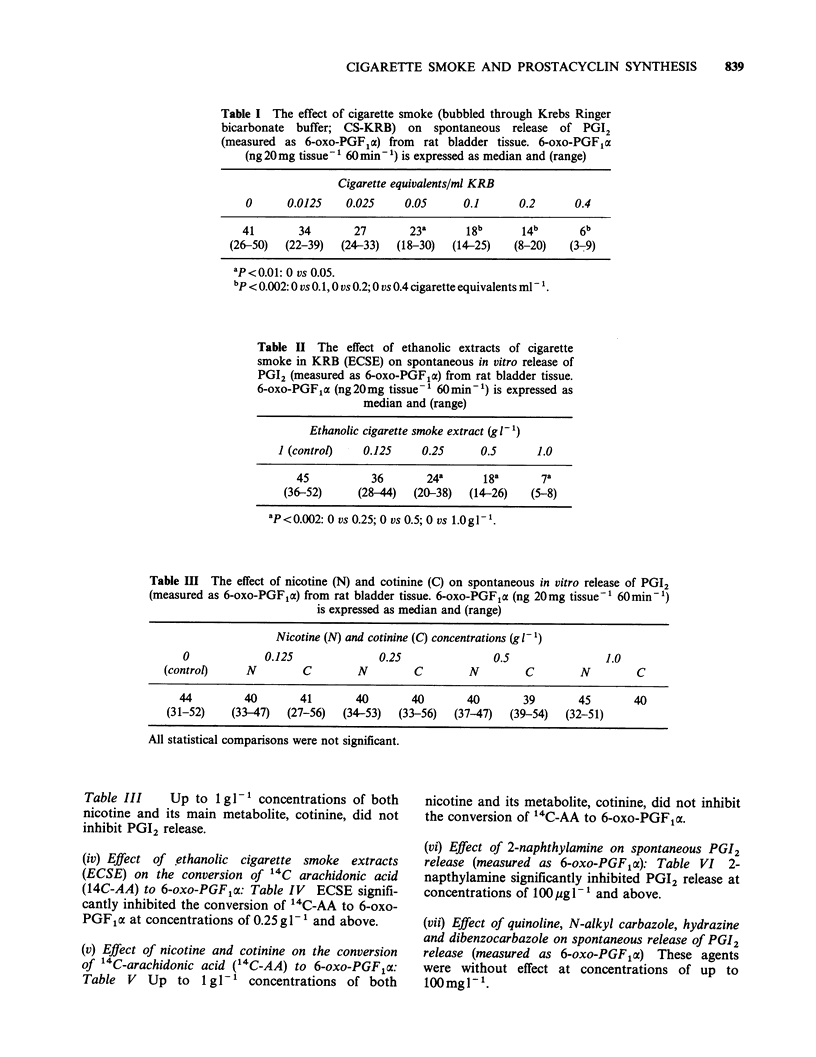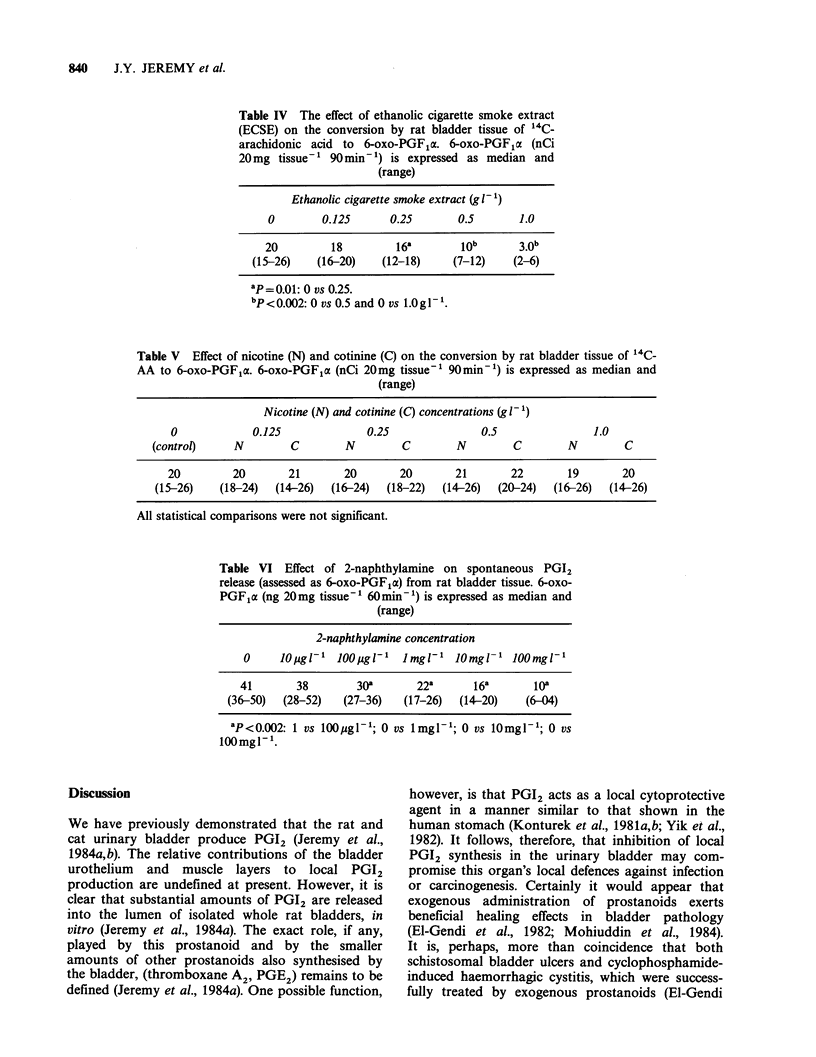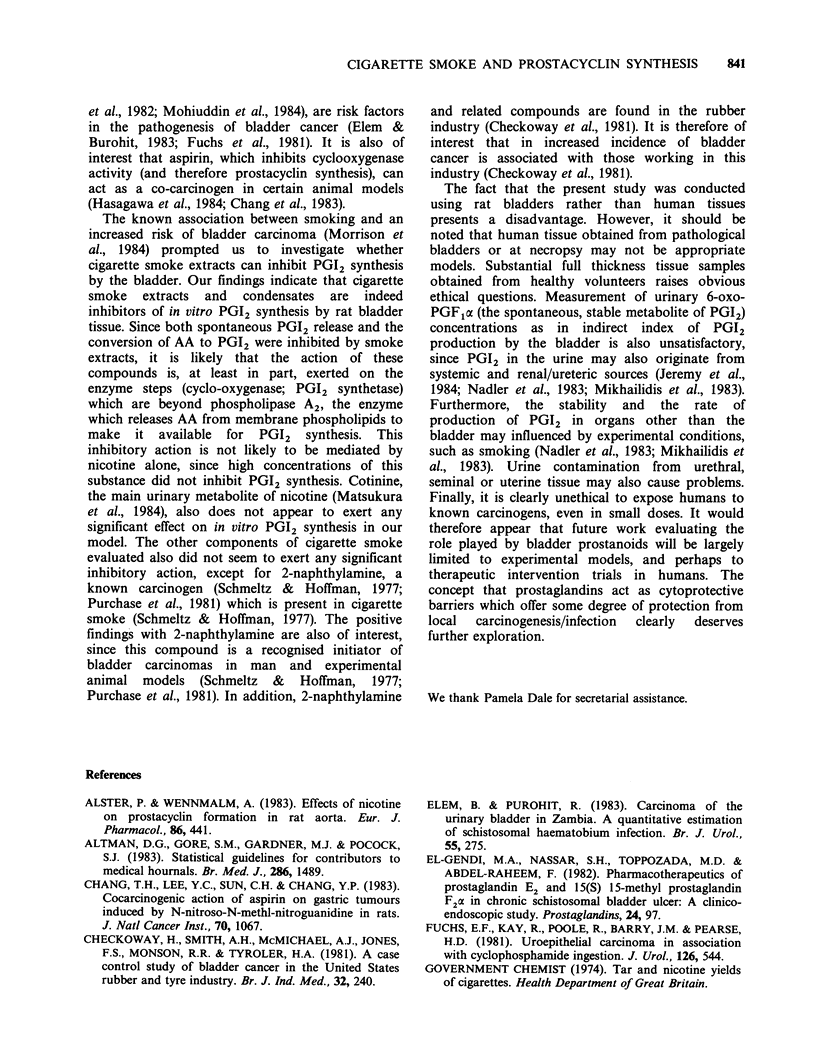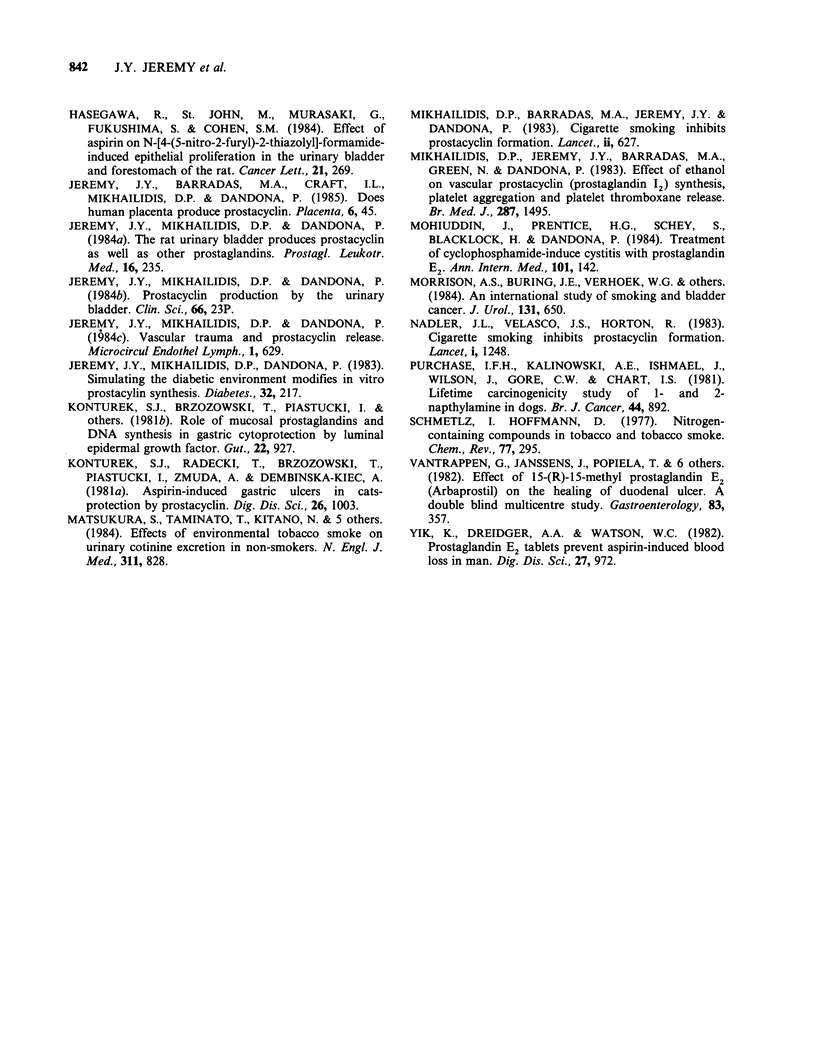# Cigarette smoke extracts inhibit prostacyclin synthesis by the rat urinary bladder.

**DOI:** 10.1038/bjc.1985.129

**Published:** 1985-06

**Authors:** J. Y. Jeremy, D. P. Mikhailidis, P. Dandona

## Abstract

Since prostacyclin (PGI2) is known to have a cytoprotective effect on epithelia, and since cigarette smoking is associated with an increased risk of bladder cancer, we investigated the possibility that nicotine, cotinine (the principal metabolite of nicotine) and other components of cigarette smoke inhibit PGI2 secretion by the urinary bladder. Using the rat urinary bladder as a model, we found that cigarette smoke extracts, but not nicotine or cotinine, inhibit in vitro PGI2 synthesis. 2-Naphthylamine, a known bladder carcinogen, was also a potent inhibitor of PGI2 synthesis by the rat bladder. It is possible that cigarette smoke and 2-napthylamine exert their carcinogenic effect partly through the inhibition of PGI2 synthesis, resulting in diminished urothelial cytoprotection.


*Br. J. Cancer* (1985), **51**, 837–842

### Cigarette smoke extracts inhibit prostacyclin synthesis by the rat urinary bladder

J.Y. Jeremy, D.P. Mikhailidis & P. Dandona

*Metabolic Unit, Department of Chemical Pathology and Human Metabolism, Royal Free Hospital and School of Medicine, London, NW3 2QG, UK.*

**Summary** Since prostacyclin ($PGI_2$) is known to have a cytoprotective effect on epithelia, and since cigarette smoking is associated with an increased risk of bladder cancer, we investigated the possibility that nicotine, cotinine (the prinicpal metabolite of nicotine) and other components of cigarette smoke inhibit $PGI_2$ secretion by the urinary bladder.

Using the rat urinary bladder as a model, we found that cigarette smoke extracts, but not nicotine or cotinine, inhibit *in vitro* $PGI_2$ synthesis. 2-Naphthylamine, a known bladder carcinogen, was also a potent inhibitor of $PGI_2$ synthesis by the rat bladder. It is possible that cigarette smoke and 2-napthylamine exert their carcinogenic effect partly through the inhibition of $PGI_2$ synthesis, resulting in diminished urothelial cytoprotection.

The mammalian bladder synthesises the prostanoid prostacyclin ($PGI_2$) in quantities comparable to vascular tissue (Jeremy *et al.*, 1984*a,b*). Although a role for $PGI_2$ in this organ has yet to be defined (Jeremy *et al.*, 1984*a*), it is possible that this prostanoid (and certain other PGs) may play a cytoprotective role in the bladder, similar to that documented for the gastrointestinal system (Konturek *et al.*, 1981*a,b*; Yik *et al.*, 1982), where certain prostanoids have even been used successfully to treat peptic ulceration (Vantrappen *et al.*, 1983). Evidence supporting the view that PGs play a cytoprotective role in the urinary bladder is provided by the finding that local administration of $PGE_2$ improves symptoms caused by schistosomal ulcers (El. Gendi *et al.*, 1982) and has also been used successfully to treat cyclophosphamide-induced haemorrhagic cystitis (Mohiuddin *et al.*, 1984). Diminution of local $PGI_2$ synthesis may therefore reduce the cytoprotective capacity of the urothelium, rendering it more susceptible to the actions of carcinogens which may be present in the urine.

Cigarette smoking is linked epidemiologically with an increased incidence of bladder cancer, although the mechanisms involved remain undefined (Morrison *et al.*, 1984). A major component of cigarette smoke, nicotine, has been shown to inhibit $PGI_2$ synthesis in vascular tissue models (Alster *et al.*, 1983). We have also shown that cigarette smoke extracts, *in vitro*, inhibit vascular $PGI_2$ synthesis (unpublished observations). We therefore studied the effect of nicotine, cotinine (the major urinary metabolite of nicotine ((Matsukura *et al.*, 1984)) and cigarette smoke extracts on *in vitro* $PGI_2$ synthesis by the rat urinary bladder. We also studied the effect of 2-naphthylamine on $PGI_2$ synthesis, since this substance is a known bladder carcinogen (Schmeltz & Hoffman, 1977).

Correspondence: P. Dandona
Received 30 November 1984; and in revised form 14 February 1985

### Material and methods

### *Preparation of cigarette smoke extracts and condensates*

The filter end of a cigarette (middle tar; approximate tar yield: 21 mg/cigarette; approximate nicotine yield: 1.3 mg/cigarette (Schmeltz & Hoffman, 1977; Government Chemist, 1974) was inserted into the end of a 10 cm length of plastic tubing. The other end of the tubing was attached to tubing in either 10 ml Krebs Ringer bicarbonate buffer (KRB), pH 7.4, or in 10 mol ethanol, contained in a flask. This system allowed us to assess both water-soluble and ethanol-soluble cigarette smoke components. Vacuum was applied to the flask through another tube so that the ignited cigarette was completely consumed (tobacco portion only) over a 5 min period, the smoke bubbling through the KRB or ethanol in the flask.

The ethanolic extract was evaporated *in vacuo* and the remaining condensate was resuspended in KRB or TRIS assay buffer (ethanolic cigarette smoke extract – ECSE). Data on ECSE is expressed as $\mu g ml^{-1}$ of condensate in buffer.

When cigarette smoke was bubbled though KRB, data are expressed as cigarette equivalents $ml^{-1}$ KRB (CS-KRB). Routinely, 1 cigarette was bubbled through 5 ml KRB, yielding 0.2 cigarette equivalents $ml^{-1}$ KRB.

© The Macmillan Press Ltd., 1985

*Preparation of nicotine and cotinine solutions*

Nicotine sulfate was obtained from Sigma Chemicals, Poole, Dorset, UK. Nicotine free base was obtained from BDH Ltd., Poole, Dorset, UK.

Cotinine (free base) was purchased from Sigma. The drugs were made up fresh in KRB prior to each experiment.

*Tissue preparation and structure of experiments*

Male Sprague-Dawley rats (200 g) were decapitated, their bladders excised and placed in chilled KRB. The bladders were cut longitudinally into two equal halves and further cut longitudinally into 6 strips with a scalpel blade on a Teflon block. Each strip was then cut into ~2 mm squares and placed in chilled KRB prior to incubations.

The following experiments were then carried out:

(*i*) *Effect of CS-KRB, ECSE, nicotine and cotinine on spontaneous* $PGI_2$ *release by bladder tissue squares* Twenty mg portions of each tissue preparation, in octuplicate, were placed in 1 ml ECSE (final concentration: 0–1 g condensate $l^{-1}$ buffer) and CS-KRB (final concentration: 0–0.4 cigarette equivalents $ml^{-1}$ KRB). Changes in buffer pH were monitored and adjusted accordingly to pH 7.4 with dilute HCl or NaOH. Bladder tissue was then incubated for 60 min in a shaking water bath (37°C). At the end of incubation, the sample was centrifuged and an aliquot of the supernatant was collected and diluted with radioimmunoassay buffer for estimation of 6-oxo-$PGF_1\alpha$ (the stable, spontaneous metabolite of $PGI_2$) concentration, using a specific radioimmunoassay (see details below).

Nicotine free base and nicotine sulphate produced identical effects and therefore the results have been summated.

*Radioimmunoassay of* 6-*oxo*-$PGF_1\alpha$ Antisera for 6-oxo-$PGF_1\alpha$ assays were purchased from Cappel Laboratories, West Chester, Pa., USA, and [$^3$H]-6-oxo-$PGF_1\alpha$ (160 Ci $mmol^{-1}$) from New England Nuclear, Boston, Ma., USA, Unlabelled 6-oxo-$PGF_1\alpha$ was a gift from Upjohn Co., Kalamzoo, Mi., USA. Assays were carried out according to protocols obtained from Cappel Laboratories (Jeremy *et al.*, 1984*a*,*c*; Mikhailidis *et al.*, 1983).

(*ii*) *Effect of carcinogens on spontaneous release of* $PGI_2$ *by bladder tissue* The known bladder carcinogen, 2-napthylamine (Schmeltz & Hoffman, 1977), was studied as in (i) above, Quinoline, N-alkyl carbazole, hydrazine, and dibenzocarbazole as tumour initiators or accelerators (Schmeltz & Hoffman, 1977), were also studied. These chemicals were purchased from BDH, Poole, Dorset, UK.

(*iii*) *Effect of ECSE, nicotine, cotinine and 2-naphtylamine on* $^{14}$*C-arachidonic acid conversion into 6-oxo-*$PGF_1\alpha$ *by rat urinary bladder tissue* Conversion of $^{14}$C-arachidonic acid ($^{14}$C-AA) into 6-oxo-$PGF_1\alpha$ was carried out as previously described (Jeremy *et al.*, 1983, 1985). Briefly, 20 mg of urinary bladder tissue were incubated, in octuplicate, in 100 $\mu$l TRIS-HCl buffer [pH 8.0; containing 1 mmol $l^{-1}$ EDTA; 150 mmol $l^{-1}$ NaCl and 250 nCi $^{14}$C-AA (52 mCi $mmol^{-1}$), New England Nuclear, Boston, Ma., USA]. ECSE, nicotine, cotinine and 2-naphthylamine were dissolved in TRIS buffer to study dose reponse relationships as described in (i) above. Following incubation, the tissue was extracted with ×2 ethanol (Aristar, BDH, Poole, UK) and pooled supernatant evaporated *in vacuo*. $^{14}$C-6-oxo-$PGF_1\alpha$ and unchanged $^{14}$C-AA were separated by thin layer chromatography (tlc) on silica gel precoated plastic plates (Merck Darmstadt, W. Germany) run in the organic phase of an ethyl acetate: 2,2,4, trimethylpentane; acetic acid; water mix (Jeremy *et al.*, 1983, 1985). The zones corresponding to 6-oxo-$PGF_1\alpha$ and AA were cut and assayed for radioactivity by liquid scintillation counting and % conversion of $^{14}$C-AA to $^{14}$C-6-oxo-$PGF_1\alpha$ was calculated (Nadler *et al.*, 1983; Chechoway *et al.*, 1983).

*Statistical analysis and presentation of data*

Data are presented as median and range (Altman *et al.*, 1983). Results in control experiments are compared with those in the presence of agents or extracts being evaluated using a non-parametric Mann-Whitney test (two-tailed) (Altman *et al.*, 1983)

### Results

(*i*) *Effect of cigarette smoke* (*bubbled through Krebs–Ringer bicarbonate buffer; CS-KRB*) *on spontaneous* in vitro *release of* $PGI_2$ (*measured as 6-oxo-*$PGF_1\alpha$): *Table I* CS-KRB was significantly ($P<0.01$) inhibitory to *in vitro* $PGI_2$ at 0.05 cigarette equivalents ml. Higher CS-KRB concentrations produced progressively more severe inhibition of $PGI_2$ release. The inhibitory trend of CS-KRB was evident at 0.0125 and 0.025 cigarette equivalents $ml^{-1}$, but this did not achieve statistical significance.

(*ii*) *Effect of ethanolic cigarette smoke extracts* (*ECSE*) *on spontaneous* in vitro $PGI_2$ *release* (*measured as 6-oxo-*$PGF_1\alpha$): *Table II* Results were very similar to those described in (i) above. The inhibitory trend was again evident at concentrations below those which showed statistical significance.

(*iii*) *The effect of nicotine and cotinine on spontaneous* in vitro *release of* $PGI_2$ (*measured as 6-oxo-*$PGF_1\alpha$):

**Table I** The effect of cigarette smoke (bubbled through Krebs Ringer bicarbonate buffer; CS-KRB) on spontaneous release of $PGI_2$ (measured as 6-oxo-$PGF_1\alpha$) from rat bladder tissue. 6-oxo-$PGF_1\alpha$ (ng 20 mg tissue$^{-1}$ 60 min$^{-1}$) is expressed as median and (range)

| *Cigarette equivalents/ml KRB* | | | | | | |
|---|---|---|---|---|---|---|
| *0* | *0.0125* | *0.025* | *0.05* | *0.1* | *0.2* | *0.4* |
| 41<br>(26–50) | 34<br>(22–39) | 27<br>(24–33) | 23[a]<br>(18–30) | 18[b]<br>(14–25) | 14[b]<br>(8–20) | 6[b]<br>(3–9) |

[a]$P<0.01$: 0 *vs* 0.05.
[b]$P<0.002$: 0 *vs* 0.1, 0 *vs* 0.2; 0 *vs* 0.4 cigarette equivalents ml$^{-1}$.

**Table II** The effect of ethanolic extracts of cigarette smoke in KRB (ECSE) on spontaneous *in vitro* release of $PGI_2$ (measured as 6-oxo-$PGF_1\alpha$) from rat bladder tissue. 6-oxo-$PGF_1\alpha$ (ng 20 mg tissue$^{-1}$ 60 min$^{-1}$) is expressed as median and (range)

| *Ethanolic cigarette smoke extract (g l$^{-1}$)* | | | | |
|---|---|---|---|---|
| *1 (control)* | *0.125* | *0.25* | *0.5* | *1.0* |
| 45<br>(36–52) | 36<br>(28–44) | 24[a]<br>(20–38) | 18[a]<br>(14–26) | 7[a]<br>(5–8) |

[a]$P<0.002$: 0 *vs* 0.25; 0 *vs* 0.5; 0 *vs* 1.0 g l$^{-1}$.

**Table III** The effect of nicotine (N) and cotinine (C) on spontaneous *in vitro* release of $PGI_2$ (measured as 6-oxo-$PGF_1\alpha$) from rat bladder tissue. 6-oxo-$PGF_1\alpha$ (ng 20 mg tissue$^{-1}$ 60 min$^{-1}$) is expressed as median and (range)

| *Nicotine (N) and cotinine (C) concentrations (g l$^{-1}$)* | | | | | | | | |
|---|---|---|---|---|---|---|---|---|
| *0* | *0.125* | | *0.25* | | *0.5* | | *1.0* | |
| *(control)* | *N* | *C* | *N* | *C* | *N* | *C* | *N* | *C* |
| 44<br>(31–52) | 40<br>(33–47) | 41<br>(27–56) | 40<br>(34–53) | 40<br>(33–56) | 40<br>(37–47) | 39<br>(39–54) | 45<br>(32–51) | 40 |

All statistical comparisons were not significant.

*Table III* Up to 1 g l$^{-1}$ concentrations of both nicotine and its main metabolite, cotinine, did not inhibit $PGI_2$ release.

(*iv*) *Effect of ethanolic cigarette smoke extracts (ECSE) on the conversion of $^{14}C$ arachidonic acid (14C-AA) to 6-oxo-$PGF_1\alpha$: Table IV* ECSE significantly inhibited the conversion of $^{14}$C-AA to 6-oxo-$PGF_1\alpha$ at concentrations of 0.25 g l$^{-1}$ and above.

(*v*) *Effect of nicotine and cotinine on the conversion of $^{14}C$-arachidonic acid ($^{14}C$-AA) to 6-oxo-$PGF_1\alpha$: Table V* Up to 1 g l$^{-1}$ concentrations of both nicotine and its metabolite, cotinine, did not inhibit the conversion of $^{14}$C-AA to 6-oxo-$PGF_1\alpha$.

(*vi*) *Effect of 2-naphthylamine on spontaneous $PGI_2$ release (measured as 6-oxo-$PGF_1\alpha$): Table VI* 2-napthylamine significantly inhibited $PGI_2$ release at concentrations of 100 μg l$^{-1}$ and above.

(*vii*) *Effect of quinoline, N-alkyl carbazole, hydrazine and dibenzocarbazole on spontaneous release of $PGI_2$ release (measured as 6-oxo-$PGF_1\alpha$)* These agents were without effect at concentrations of up to 100 mg l$^{-1}$.

**Table IV** The effect of ethanolic cigarette smoke extract (ECSE) on the conversion by rat bladder tissue of $^{14}C$-arachidonic acid to 6-oxo-$PGF_1\alpha$. 6-oxo-$PGF_1\alpha$ (nCi 20 mg tissue$^{-1}$ 90 min$^{-1}$) is expressed as median and (range)

| *Ethanolic cigarette smoke extract* ($g\,l^{-1}$) | | | | |
|---|---|---|---|---|
| *0* | *0.125* | *0.25* | *0.5* | *1.0* |
| 20 (15–26) | 18 (16–20) | 16[a] (12–18) | 10[b] (7–12) | 3.0[b] (2–6) |

[a]$P = 0.01$: 0 *vs* 0.25.
[b]$P < 0.002$: 0 *vs* 0.5 and 0 *vs* 1.0 g l$^{-1}$.

**Table V** Effect of nicotine (N) and cotinine (C) on the conversion by rat bladder tissue of $^{14}C$-AA to 6-oxo-$PGF_1\alpha$. 6-oxo-$PGF_1\alpha$ (nCi 20 mg tissue$^{-1}$ 90 min$^{-1}$) is expressed as median and (range)

| *Nicotine (N) and cotinine (C) concentrations* ($g\,l^{-1}$) | | | | | | | | |
|---|---|---|---|---|---|---|---|---|
| *0* | *0.125* | | *0.25* | | *0.5* | | *1.0* | |
| *(control)* | *N* | *C* | *N* | *C* | *N* | *C* | *N* | *C* |
| 20 (15–26) | 20 (18–24) | 21 (14–26) | 20 (16–24) | 20 (18–22) | 21 (14–26) | 22 (20–24) | 19 (16–26) | 20 (14–26) |

All statistical comparisons were not significant.

**Table VI** Effect of 2-naphthylamine on spontaneous $PGI_2$ release (assessed as 6-oxo-$PGF_1\alpha$) from rat bladder tissue. 6-oxo-$PGF_1\alpha$ (ng 20 mg tissue$^{-1}$ 60 min$^{-1}$) is expressed as median and (range)

| *2-naphthylamine concentration* | | | | | |
|---|---|---|---|---|---|
| *0* | *10* $\mu g\,l^{-1}$ | *100* $\mu g\,l^{-1}$ | *1* $mg\,l^{-1}$ | *10* $mg\,l^{-1}$ | *100* $mg\,l^{-1}$ |
| 41 (36–50) | 38 (28–52) | 30[a] (27–36) | 22[a] (17–26) | 16[a] (14–20) | 10[a] (6–04) |

[a]$P < 0.002$: 1 *vs* 100 µg l$^{-1}$; 0 *vs* 1 mg l$^{-1}$; 0 *vs* 10 mg l$^{-1}$; 0 *vs* 100 mg l$^{-1}$.

### Discussion

We have previously demonstrated that the rat and cat urinary bladder produce $PGI_2$ (Jeremy *et al.*, 1984*a,b*). The relative contributions of the bladder urothelium and muscle layers to local $PGI_2$ production are undefined at present. However, it is clear that substantial amounts of $PGI_2$ are released into the lumen of isolated whole rat bladders, *in vitro* (Jeremy *et al.*, 1984*a*). The exact role, if any, played by this prostanoid and by the smaller amounts of other prostanoids also synthesised by the bladder, (thromboxane $A_2$, $PGE_2$) remains to be defined (Jeremy *et al.*, 1984*a*). One possible function, however, is that $PGI_2$ acts as a local cytoprotective agent in a manner similar to that shown in the human stomach (Konturek *et al.*, 1981*a,b*; Yik *et al.*, 1982). It follows, therefore, that inhibition of local $PGI_2$ synthesis in the urinary bladder may compromise this organ's local defences against infection or carcinogenesis. Certainly it would appear that exogenous administration of prostanoids exerts beneficial healing effects in bladder pathology (El-Gendi *et al.*, 1982; Mohiuddin *et al.*, 1984). It is, perhaps, more than coincidence that both schistosomal bladder ulcers and cyclophosphamide-induced haemorrhagic cystitis, which were successfully treated by exogenous prostanoids (El-Gendi

*et al.*, 1982; Mohiuddin *et al.*, 1984), are risk factors in the pathogenesis of bladder cancer (Elem & Burohit, 1983; Fuchs *et al.*, 1981). It is also of interest that aspirin, which inhibits cyclooxygenase activity (and therefore prostacyclin synthesis), can act as a co-carcinogen in certain animal models (Hasagawa *et al.*, 1984; Chang *et al.*, 1983).

The known association between smoking and an increased risk of bladder carcinoma (Morrison *et al.*, 1984) prompted us to investigate whether cigarette smoke extracts can inhibit $PGI_2$ synthesis by the bladder. Our findings indicate that cigarette smoke extracts and condensates are indeed inhibitors of *in vitro* $PGI_2$ synthesis by rat bladder tissue. Since both spontaneous $PGI_2$ release and the conversion of AA to $PGI_2$ were inhibited by smoke extracts, it is likely that the action of these compounds is, at least in part, exerted on the enzyme steps (cyclo-oxygenase; $PGI_2$ synthetase) which are beyond phospholipase $A_2$, the enzyme which releases AA from membrane phospholipids to make it available for $PGI_2$ synthesis. This inhibitory action is not likely to be mediated by nicotine alone, since high concentrations of this substance did not inhibit $PGI_2$ synthesis. Cotinine, the main urinary metabolite of nicotine (Matsukura *et al.*, 1984), also does not appear to exert any significant effect on *in vitro* $PGI_2$ synthesis in our model. The other components of cigarette smoke evaluated also did not seem to exert any significant inhibitory action, except for 2-naphthylamine, a known carcinogen (Schmeltz & Hoffman, 1977; Purchase *et al.*, 1981) which is present in cigarette smoke (Schmeltz & Hoffman, 1977). The positive findings with 2-naphthylamine are also of interest, since this compound is a recognised initiator of bladder carcinomas in man and experimental animal models (Schmeltz & Hoffman, 1977; Purchase *et al.*, 1981). In addition, 2-naphthylamine and related compounds are found in the rubber industry (Checkoway *et al.*, 1981). It is therefore of interest that in increased incidence of bladder cancer is associated with those working in this industry (Checkoway *et al.*, 1981).

The fact that the present study was conducted using rat bladders rather than human tissues presents a disadvantage. However, it should be noted that human tissue obtained from pathological bladders or at necropsy may not be appropriate models. Substantial full thickness tissue samples obtained from healthy volunteers raises obvious ethical questions. Measurement of urinary 6-oxo-$PGF_1\alpha$ (the spontaneous, stable metabolite of $PGI_2$) concentrations as in indirect index of $PGI_2$ production by the bladder is also unsatisfactory, since $PGI_2$ in the urine may also originate from systemic and renal/ureteric sources (Jeremy *et al.*, 1984; Nadler *et al.*, 1983; Mikhailidis *et al.*, 1983). Furthermore, the stability and the rate of production of $PGI_2$ in organs other than the bladder may influenced by experimental conditions, such as smoking (Nadler *et al.*, 1983; Mikhailidis *et al.*, 1983). Urine contamination from urethral, seminal or uterine tissue may also cause problems. Finally, it is clearly unethical to expose humans to known carcinogens, even in small doses. It would therefore appear that future work evaluating the role played by bladder prostanoids will be largely limited to experimental models, and perhaps to therapeutic intervention trials in humans. The concept that prostaglandins act as cytoprotective barriers which offer some degree of protection from local carcinogenesis/infection clearly deserves further exploration.

We thank Pamela Dale for secretarial assistance.

**References**

ALSTER, P. & WENNMALM, A. (1983). Effects of nicotine on prostacyclin formation in rat aorta. *Eur. J. Pharmacol.*, **86**, 441.

ALTMAN, D.G., GORE, S.M., GARDNER, M.J. & POCOCK, S.J. (1983). Statistical guidelines for contributors to medical hournals. *Br. Med. J.*, **286**, 1489.

CHANG, T.H., LEE, Y.C., SUN, C.H. & CHANG, Y.P. (1983). Cocarcinogenic action of aspirin on gastric tumours induced by N-nitroso-N-methl-nitroguanidine in rats. *J. Natl Cancer Inst.*, **70**, 1067.

CHECKOWAY, H., SMITH, A.H., McMICHAEL, A.J., JONES, F.S., MONSON, R.R. & TYROLER, H.A. (1981). A case control study of bladder cancer in the United States rubber and tyre industry. *Br. J. Ind. Med.*, **32**, 240.

ELEM, B. & PUROHIT, R. (1983). Carcinoma of the urinary bladder in Zambia. A quantitative estimation of schistosomal haematobium infection. *Br. J. Urol.*, **55**, 275.

EL-GENDI, M.A., NASSAR, S.H., TOPPOZADA, M.D. & ABDEL-RAHEEM, F. (1982). Pharmacotherapeutics of prostaglandin $E_2$ and 15(S) 15-methyl prostaglandin $F_2\alpha$ in chronic schistosomal bladder ulcer: A clinico-endoscopic study. *Prostaglandins*, **24**, 97.

FUCHS, E.F., KAY, R., POOLE, R., BARRY, J.M. & PEARSE, H.D. (1981). Uroepithelial carcinoma in association with cyclophosphamide ingestion. *J. Urol.*, **126**, 544.

GOVERNMENT CHEMIST (1974). Tar and nicotine yields of cigarettes. *Health Department of Great Britain.*

HASEGAWA, R., St. JOHN, M., MURASAKI, G., FUKUSHIMA, S. & COHEN, S.M. (1984). Effect of aspirin on N-[4-(5-nitro-2-furyl)-2-thiazolyl]-formamide-induced epithelial proliferation in the urinary bladder and forestomach of the rat. *Cancer Lett.*, **21**, 269.

JEREMY, J.Y., BARRADAS, M.A., CRAFT, I.L., MIKHAILIDIS, D.P. & DANDONA, P. (1985). Does human placenta produce prostacyclin. *Placenta*, **6**, 45.

JEREMY, J.Y., MIKHAILIDIS, D.P. & DANDONA, P. (1984*a*). The rat urinary bladder produces prostacyclin as well as other prostaglandins. *Prostagl. Leukotr. Med.*, **16**, 235.

JEREMY, J.Y., MIKHAILIDIS, D.P. & DANDONA, P. (1984*b*). Prostacyclin production by the urinary bladder. *Clin. Sci.*, **66**, 23P.

JEREMY, J.Y., MIKHAILIDIS, D.P. & DANDONA, P. (1984*c*). Vascular trauma and prostacyclin release. *Microcircul Endothel Lymph.*, **1**, 629.

JEREMY, J.Y., MIKHAILIDIS, D.P., DANDONA, P. (1983). Simulating the diabetic environment modifies in vitro prostacylin synthesis. *Diabetes.*, **32**, 217.

KONTUREK, S.J., BRZOZOWSKI, T., PIASTUCKI, I. & others. (1981*b*). Role of mucosal prostaglandins and DNA synthesis in gastric cytoprotection by luminal epidermal growth factor. *Gut.*, **22**, 927.

KONTUREK, S.J., RADECKI, T., BRZOZOWSKI, T., PIASTUCKI, I., ZMUDA, A. & DEMBINSKA-KIEC, A. (1981*a*). Aspirin-induced gastric ulcers in cats-protection by prostacyclin. *Dig. Dis. Sci.*, **26**, 1003.

MATSUKURA, S., TAMINATO, T., KITANO, N. & 5 others. (1984). Effects of environmental tobacco smoke on urinary cotinine excretion in non-smokers. *N. Engl. J. Med.*, **311**, 828.

MIKHAILIDIS, D.P., BARRADAS, M.A., JEREMY, J.Y. & DANDONA, P. (1983). Cigarette smoking inhibits prostacyclin formation. *Lancet.*, **ii**, 627.

MIKHAILIDIS, D.P., JEREMY, J.Y., BARRADAS, M.A., GREEN, N. & DANDONA, P. (1983). Effect of ethanol on vascular prostacyclin (prostaglandin $I_2$) synthesis, platelet aggregation and platelet thromboxane release. *Br. Med. J.*, **287**, 1495.

MOHIUDDIN, J., PRENTICE, H.G., SCHEY, S., BLACKLOCK, H. & DANDONA, P. (1984). Treatment of cyclophosphamide-induce cystitis with prostaglandin $E_2$. *Ann. Intern. Med.*, **101**, 142.

MORRISON, A.S., BURING, J.E., VERHOEK, W.G. & others. (1984). An international study of smoking and bladder cancer. *J. Urol.*, **131**, 650.

NADLER, J.L., VELASCO, J.S., HORTON, R. (1983). Cigarette smoking inhibits prostacyclin formation. *Lancet*, **i**, 1248.

PURCHASE, I.F.H., KALINOWSKI, A.E., ISHMAEL, J., WILSON, J., GORE, C.W. & CHART, I.S. (1981). Lifetime carcinogenicity study of 1- and 2-napthylamine in dogs. *Br. J. Cancer*, **44**, 892.

SCHMETLZ, I. HOFFMANN, D. (1977). Nitrogen-containing compounds in tobacco and tobacco smoke. *Chem., Rev.*, **77**, 295.

VANTRAPPEN, G., JANSSENS, J., POPIELA, T. & 6 others. (1982). Effect of 15-(R)-15-methyl prostaglandin $E_2$ (Arbaprostil) on the healing of duodenal ulcer. A double blind multicentre study. *Gastroenterology*, **83**, 357.

YIK, K., DREIDGER, A.A. & WATSON, W.C. (1982). Prostaglandin $E_2$ tablets prevent aspirin-induced blood loss in man. *Dig. Dis. Sci.*, **27**, 972.